# Carbon Dioxide vs. Air Insufflation for Pediatric Gastrointestinal Endoscopy: A Systematic Review and Meta-Analysis of Randomized Controlled Trials

**DOI:** 10.3389/fped.2021.610066

**Published:** 2021-02-09

**Authors:** Chunwang Ji, Xue Liu, Peng Huang

**Affiliations:** ^1^Grade 2017, Queen Mary Institute, Nanchang University, Nanchang, China; ^2^Shandong University of Traditional Chinese Medicine, Jinan, China; ^3^Jiangxi Province Key Laboratory of Preventive Medicine, School of Public Health, Nanchang University, Nanchang, China

**Keywords:** Carbon dioxide, gastrointestinal pain, children, colonoscopy, endoscopy

## Abstract

**Background:** Carbon dioxide (CO_2_) insufflation during gastrointestinal (GI) endoscopic procedures has gained popularity in adults. However, its utility in pediatric patients is not known. The current review aimed to compare the efficacy of CO_2_ vs. air insufflation for GI endoscopic procedures in pediatric patients.

**Methods:** The electronic databases of PubMed, Embase, Scopus, and CENTRAL were searched from the inception of databases to 15th August 2020.

**Results:** All randomized controlled trials (RCTs) comparing CO_2_ vs. air insufflation for GI endoscopic procedures in pediatric patients were eligible for inclusion. Five RCTs were identified. Pooled analysis of data from 226 patients in the CO_2_ group and 224 patients in the air group revealed that patients receiving CO_2_ insufflation were at a lower odds of experiencing postoperative pain as compared to those undergoing the procedure with air (OR: 0.40; 95% CI: 0.19, 0.87; *I*^2^ = 62%; *p* = 0.02). Descriptive analysis indicated no difference in the two groups for abdominal distention after the procedure. Two trials reported elevated CO_2_ in the study group but without any pulmonary complications. Bloating was reported by two studies and both reported significantly less bloating in the CO_2_ group.

**Conclusion:** Our study indicates that the incidence of pain may be reduced with the use of CO_2_ insufflation in pediatric GI endoscopies without a significant risk of adverse events. However, current evidence is from a limited number of trials and not strong to recommend a routine of CO_2_ in pediatric gastroenterology practice. Further high-quality RCTs are required to supplement current evidence.

## Introduction

Endoscopy is a common procedure in a pediatric gastroenterology practice, with current technology permitting examination of all patients from infants to adults. Optimal intestinal distention is extremely essential for proper examination during endoscopy whether it is esophagogastroduodenoscopy (EGD) or colonoscopy (1). Traditionally, air has been used for insufflation of the gastrointestinal (GI) tract. However, owing to its poor intestinal resorption, it frequently leads to post-operative bowel distention and bloating (2). Pain and discomfort after the procedure is frequently attributed to the residual bowel gas (3).

In the 1950s, carbon dioxide (CO_2_) was first proposed as an insufflating agent for adult colonoscopies and rigid sigmoidoscopies (4). Since the absorption rate of CO_2_ is 160 times that of nitrogen and 13 times that of oxygen, the residual gas after endoscopy with CO_2_ insufflation is significantly less (5, 6). Over the last two decades, several studies have confirmed the efficacy and safety of CO_2_ insufflation as compared to air in adult patients especially for colonoscopy and enteroscopy. In a 2016 systematic review and meta-analysis, Memon et al. (7) have demonstrated that the use of CO_2_ insufflation in adult colonoscopies significantly reduced intra-operative abdominal pain with effects lasting up to 24 h. These results are further corroborated by a recent meta-analysis of Rogers et al. (8), who have found that adult patients undergoing colonoscopy with CO_2_ insufflation have significantly reduced pain scores with less distention, bloating, and flatulence. Aquino et al. (9) in a meta-analysis have demonstrated that the use of CO_2_ insufflation during enteroscopy significantly reduces pain at 1 and 4 h after the procedure.

Pediatric patients routinely undergo EGD or colonoscopy as a diagnostic or therapeutic procedure (10–14). However, with continuous research, the indications for EGD or colonoscopy in pediatric patients is constantly evolving. In children, EGD is being increasingly used not only for diagnosis of eosinophilic esophagitis but also for surveillance and to assess disease activity post-treatment (15). On the other hand, with increased accuracy of serological tests, endoscopic investigations can be avoided for patients with celiac disease (16). Despite numerous clinical trials and reviews on the use of CO_2_ insufflation for endoscopy in adults (7, 9, 17, 18), data concerning pediatric patients is scarce. Over the past few years, studies have evaluated the benefits of CO_2_ insufflation during GI endoscopy in pediatric patients. However, to the best of our knowledge, no attempt has been made to synthesize data from these studies to provide level-1 evidence on the use of CO_2_ insufflation for pediatric patients. Therefore, the purpose of this review was to conduct a systematic literature search and pool data comparing the efficacy of CO_2_ insufflation vs. air insufflation for GI endoscopic procedures in pediatric patients.

## Materials and Methods

### Search Strategy

The authors designed and implemented this review adhering to the guidelines of the PRISMA statement (Preferred Reporting Items for Systematic Reviews and Meta-analyses) (19) and the Cochrane Handbook for Systematic Reviews of Intervention (20), except for protocol registration. The electronic databases of PubMed, Embase, Scopus, and CENTRAL were searched by two reviewers independently. Search limits were from inception of databases to 15th August 2020. For the search, we used a combination of MeSH terms and free-text keywords. The terms “carbon dioxide,” “CO_2_,” “air,” “colonoscopy,” “endoscopy,” “enteroscopy,” “insufflation,” “pediatric,” “children,” and “pain” were used in different combinations. The reviewers screened the search results initially by their titles and abstracts for each database. After identifying potentially pertinent articles, full texts of the articles were extracted. Both the reviewers assessed individual articles based on the inclusion and exclusion criteria. Any disagreements were resolved by discussion. After screening, the bibliography of included studies and review articles on the subject were hand searched for any missed references.

### Inclusion Criteria

Only randomized controlled trials (RCTs) were eligible to be included in the review. We further defined the inclusion criteria based on the PICO (Population, Intervention, Comparison, Outcome) framework as follows:- *Population*: studies conducted on pediatric patients undergoing GI endoscopy. The *intervention* was to be CO_2_ insufflation for visualization during the procedure compared (*Comparison*) to air. Included studies were to report at least one of the following *outcomes:* pain, abdominal distention, and elevated EtCO_2_. No language restriction was placed. We excluded studies on adults, non-RCTs, retrospective studies, single-arm studies, and studies not reporting relevant data.

### Data Extraction

After mutual agreement on the inclusion of studies, data were extracted by two reviewers independently. Data regarding authors, publication year, study type, demographic details, body mass index (BMI), sample size, patients with pre-procedural abdominal pain, duration of the procedure, anesthesia protocol, and study outcomes were extracted. The primary outcome of the interest of our analysis was pain after the procedure. The secondary outcomes were abdominal distention and elevated EtCO_2_. Any other outcomes reported by the included studies were reported also descriptively.

### Risk of Bias Assessment

The Cochrane Collaboration risk assessment tool was used for assessing the quality of included RCTs (20). Two reviewers independently assessed each study. The following seven domains were used for quality assessment: random sequence generation, allocation concealment, blinding of participants and personnel, blinding of outcome assessment, incomplete outcome data, and selective reporting. The study was judged to have a “high,” “unclear,” or “low” risk of bias for each domain. Any disagreements were resolved by discussion.

### Statistical Analysis

Meta-analysis was conducted in at least three trials reported similar outcomes, otherwise, a descriptive analysis was carried out. “Review Manager” (RevMan, version 5.3; Nordic Cochrane Centre [Cochrane Collaboration], Copenhagen, Denmark; 2014) was used for the meta-analysis. Outcome data was fed into meta-analysis software and cross-checked for correctness. Since included studies reported the presence/absence of pain in the postoperative period, we calculated Odds ratios (OR) with 95% confidence intervals (CI). We used a random-effects model to calculate the pooled effect size for all our analysis. Heterogeneity was assessed using the *I*^2^ statistic. *I*^2^ values of 25–50% represented low, values of 50–75% medium, and more than 75% represented substantial heterogeneity. We also assessed the influence of each study on the pooled OR using a sensitivity analysis. Due to the inclusion of fewer than 10 studies per meta-analysis, funnel plots were not used to assess publication bias.

## Results

**Figure 1:**
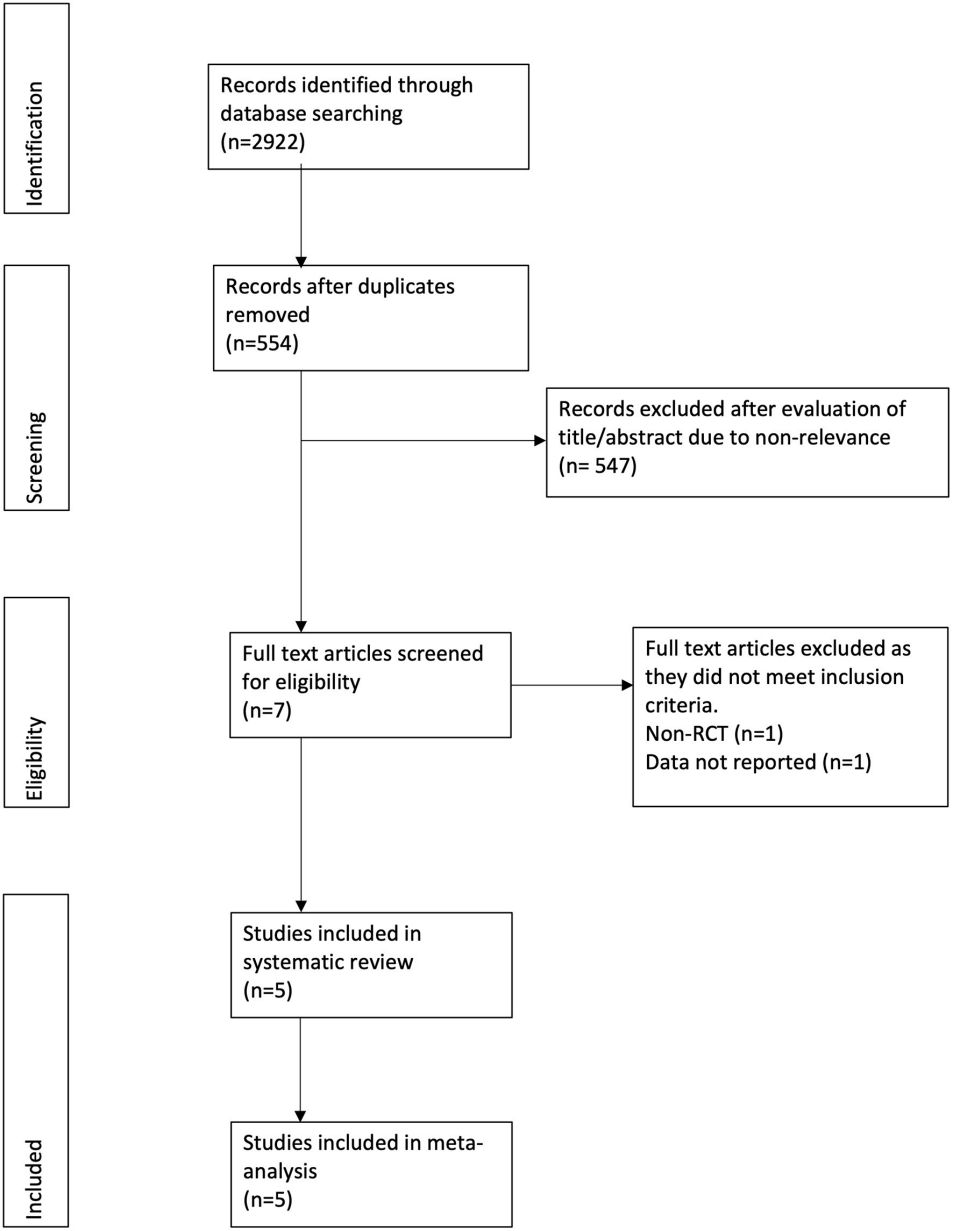
Study flow chart.

The study flow chart is presented in Figure 1. A total of seven articles were assessed of which two were excluded (21, 22). Five studies (23–27) met the inclusion criteria and were included in the review. Details of the included studies are presented in Table 1. All were RCTs mostly conducted in the USA. In the studies of Dike et al. (27) and Kresz et al. (24) upper GI endoscopies were carried out. The sample size of the studies varied from 20 to 91 patients per arm. The definition of pediatric patients varied in the studies with one trial (27) including patients <21 years of age while the remaining including patients <18 years of age. Abdominal pain as an indication for the procedure was not significantly different between the CO_2_ and air groups in any trial. The duration of the procedure was reported by three studies (23, 24, 26) with no statistically significant differences between the two groups.

**Table 1:** Details of included studies.

**References**	**Location**	**Procedure**	**Sample size**		**Age** ** (years)**		**Male** ** gender (%)**		**BMI** ** (kg/m**^****2****^**)**		**Upper GI** ** endoscopy (%)**		**Abdominal** ** pain (%)**		**Duration of** ** procedure (min)**		**Anesthesia**	**Scale for pain scores**
			**CO_**2**_**	**Air**	**CO_**2**_**	**Air**	**CO_**2**_**	**Air**	**CO_**2**_**	**Air**	**CO_**2**_**	**Air**	**CO_**2**_**	**Air**	**CO_**2**_**	**Air**		
Dike et al. (27)	USA	Upper GI endoscopy and colonoscopy	91	89	12.8 (1.1–20.4)^*^	13.7 (1.4–20.8)^*^	48	39	0.16 (−4.96,2.61)^∧^	−0.03 (−3.79, 2.48)	48^$^	49^$^	48	53	NR	NR	Continuous propofol infusion without advanced airway (Occasionally GA used)	Pain on FLACC/Faces pain scale and GPPP scale
Dharmaraj et al. (26)	USA	Colonoscopy	48	52	13.8 ± 2.8	15.5 ± 3	54.2	44.2	20.8 (17.1–23.4)^*^	20.6 (18–24.4)^*^	–	–	12.8	24.2	28.79 ± NR	28.17 ± NR	Propofol for induction with combination of nitrous oxide, sevoflurane, propofol for deep sedation	VAS
Kresz et al. (24)	Germany	Upper GI endoscopy and Colonoscopy	39	34	13.7 ± 3.2	12.7 ± 2.7	57.4	52.9	19.5 ± 3.5	19 ± 2.5	56.4^#^	61.8^#^	33.3	50	31.3 ± 13. 2	31.5 ± 16.7	Sedation using midazolam and propofol	VAS/Faces pain scale/colored analog scale
Thornhill et al. (25)	USA	Colonoscopy	20	20	5–18		40	55	NR	NR	–	–	45	55	NR	NR	GA	VAS/Faces pain scale
Homan et al. (23)	USA	Upper GI endoscopy and Colonoscopy	38	38	13.7 ± 3.8	13.2 ± 3.3	45	50	19.7 ± 3.4	19.3 ± 3.1	76^#^	84^#^	31.5		23.5 ± 11.7	22.2 ± 8.2	Sedation using ketamine and midazolam	NRS

*RCT, randomized controlled trial; FLACC, face legs activity cry and ability to be consoled; GPPP, global parent perception of pain; VAS, visual analog scale; NRS, numerical rating scale; GI, gastrointestinal; GA, general anesthesia*.

∧ *z-score, median (range)*.

* *Median (Interquartile range)*.

# *All concomitant endoscopies*.

$ *Exclusive upper GI endoscopies*.

**Figure 2:**
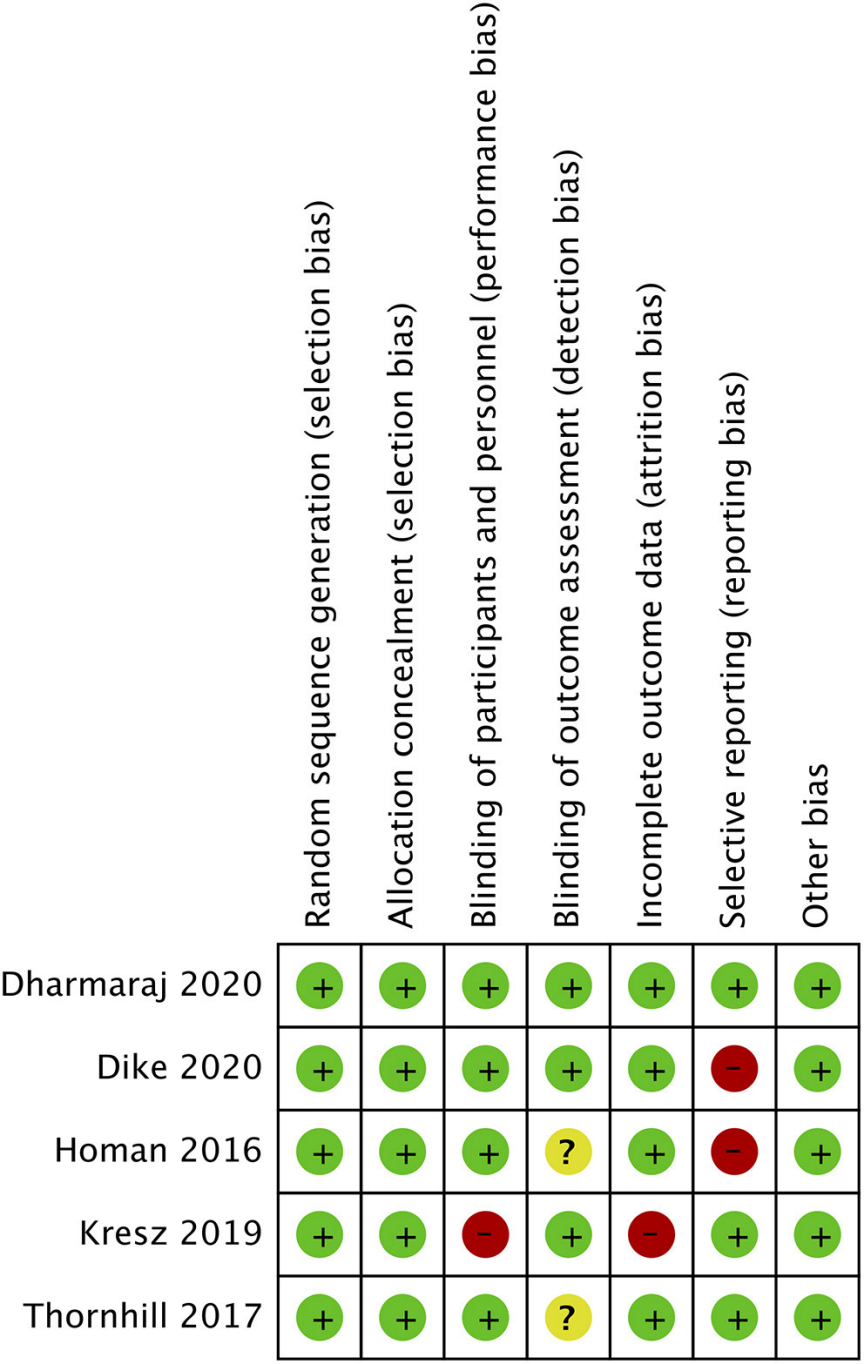
Risk of bias analysis. Red circle, high risk of bias; Yellow circle, unclear risk of bias; Green circle, low risk of bias.

The risk of bias analysis of included studies is presented in Figure 2. All trials had a low risk of bias for randomization, allocation concealment, and blinding of participants. In the study of Kresz et al. (24), the operators were no blinded. Three trials (24, 26, 27) mentioned blinding of outcomes assessment. Complete outcome data (abdominal distention) was not reported in two studies (23, 27) and hence were marked with a high risk of bias for reporting bias.

### Outcomes

**Figure 3:**
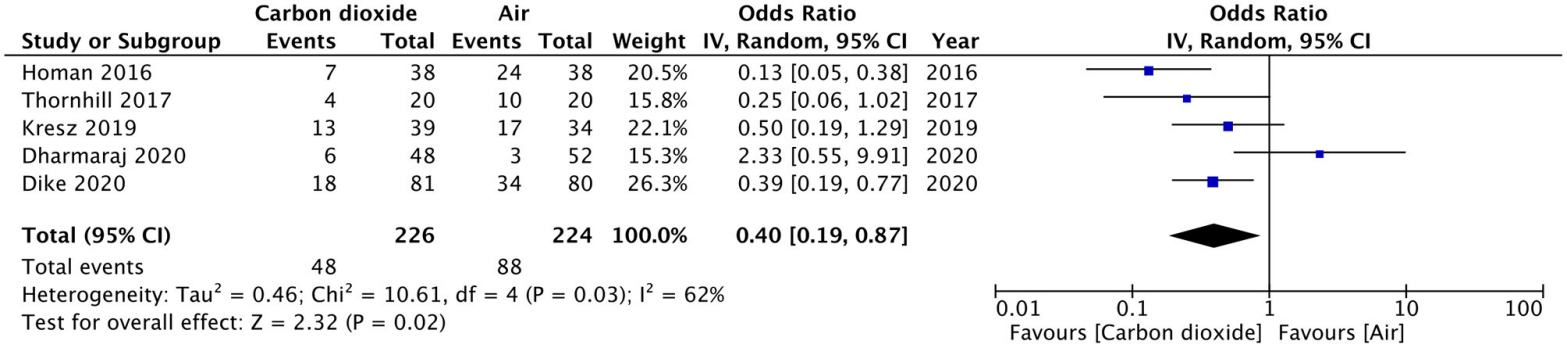
Meta-analysis of incidence of pain after pediatric GI endoscopy with CO_2_ and air.

Pain after the procedure was assessed by all included studies. All trials reported the number of patients experiencing pain after the procedure as a dichotomous variable. Pooled analysis of data from 226 patients in the CO_2_ group and 224 patients in the air group revealed that patients receiving CO_2_ insufflation were at a lower odds of experiencing postoperative pain as compared to those undergoing the procedure with air (OR: 0.40; 95% CI: 0.19, 0.87; *I*^2^ = 62%; *p* = 0.02; Figure 3). The results of the sensitivity analysis are presented in Table 2. Except for the trial of Dharmaraj et al. (26), on the exclusion of any of the remaining studies, the results were non-significant. Since pain scores as mean and standard deviation were not coherently reported by the included trials, a meta-analysis for the same could not be carried out. Similarly, data on abdominal distention and elevated EtCO_2_ were not sufficiently reported and the results were analyzed qualitatively.

**Table 2:** Results of sensitivity analysis.

**Excluded study**	**Effect size**
Dike et al. (27)	OR: 0.42; 95% CI: 0.14, 1.28; *I*^2^ = 72%; *p* = 0.13
Dharmaraj et al. (26)	OR: 0.31; 95% CI: 0.18, 0.54; *I*^2^ = 25%; *p* < 0.0001
Kresz et al. (24)	OR: 0.39; 95% CI: 0.14, 1.07; *I*^2^ = 71%; *p* = 0.07
Thornhill et al. (25)	OR: 0.45; 95% CI: 0.18, 1.11; *I*^2^ = 71%; *p* = 0.08
Homan et al. (23)	OR: 0.53; 95% CI: 0.25, 1.10; *I*^2^ = 48%; *p* = 0.09

*OR, odds ratio; CI confidence interval*.

Table 3 presents a descriptive analysis of all outcomes in the included studies. For pain scores, only Dike et al. (27) and Kresz et al. (24) did not report a statistically significant difference between the CO_2_ and air groups. However, Dike et al. (27) reported a significantly lower number of patients experiencing no pain, and Kresz et al. (24) found overall lower pain scores in the CO_2_ group. Four of the five trials evaluated the degree of abdominal distention in study groups. Dharmaraj et al. (26) reported significant distention in the air group while the remaining trials (23, 24, 27) reported no differences between the groups. Maximum EtCO_2_ levels were significantly higher in the CO_2_ group in two trials (26, 27). However, none of the studies reported any pulmonary complications with elevated CO_2_ in the study group. Bloating was measured in two trials (24, 27) and both reported significantly less bloating in the CO_2_ group.

**Table 3:** Descriptive analysis of outcomes in included studies.

**References**	**Outcome**	**Results**
Dike et al. (27)	Pain on FLACC	No statistical significant differences between the two groups in recovery
	Pain on GPPP	No statistical significant differences between the two groups in recovery and at home
	Pain on faces scale	Significantly lower number of patients with no pain in recovery in the CO_2_ group
	Abdominal distention	No statistical significant differences between the two groups at the end of the procedure, at discharge from recovery and at home
	Bloating	Significantly less in the CO_2_ group
	Flatulence	Significantly less in the CO_2_ group
	Belching	Significantly more in the CO_2_ group
	Elevated EtCO_2_	Significantly higher number of episodes of transient hypercarbia (>60 mmHg, <1 min) in the CO_2_ group. Two patients in the CO_2_ group experienced sustained hypercarbia (>60 mmHg, ≥5 min)
Dharmaraj et al. (26)	Pain	Significantly lower pain in patients in the CO_2_ group
	Pain medications in recovery	No statistical significant differences between the two groups
	Abdominal distention	Significantly increased abdominal distention at the end of the procedure in the air group
	Elevated EtCO_2_	Maximum EtCO_2_ values during the procedure significantly higher in the CO_2_ group. No adverse events related to elevated EtCO_2_ noted up to 72 h after the procedure
	Time to discharge	No statistical significant differences between the two groups
Kresz et al. (24)	Pain	Lower pain scores in the CO_2_ group at 15 min, 1, 3, and 24 h but results were not statistically significant
	Additional narcotics during procedure	Significantly higher number of patients required narcotics in the air group
	Abdominal distention	No statistical significant differences between the two groups at 5 min at 60 min after the procedure
	Bloating	Significantly less in the CO_2_ group
	Elevated PtCO_2_	No statistical significant differences between the two groups
		No statistical significant differences between the two groups
Thornhill et al. (25)	Pain	Significantly lower pain in patients in the CO_2_ group at 1 h but not at 6 and 24 h
	Elevated EtCO_2_	No statistical significant differences between the two groups at cecal intubation, end of procedure and 10 min after procedure. No pulmonary complications in either groups
Homan et al. (23)	Pain	Significantly lower pain in patients in the CO_2_ group at 2 and 4 h
	Abdominal distention	No statistical significant differences between the two groups at 10 min, 2 and 4 h after colonoscopy

*EtCO_2_, end tidal CO_2_; PtCO2, percutaneous CO_2;_ FLACC, face, legs, activity, cry, and ability to be consoled; GPPP, global parent perception of pain*.

## Discussion

The results of our review indicate that the use of CO_2_ insufflation in pediatric GI endoscopy procedures may result in a lower incidence of post-procedural pain. Descriptive analysis indicates that there is no difference in abdominal distention after the procedure with the use of either CO_2_ or air. Bloating may be less with CO_2_. Further, there may not be any increase in complications with CO_2_ use.

One of the important differences between adult and pediatric upper GI endoscopies and colonoscopies is the use of deep sedation during pediatric procedures. According to Thakkar et al. (28, 29), 54% of pediatric EGDs are carried out under general anesthesia (GA) while 46% of the procedures take place under intravenous sedation. On the other hand, approximately half of colonoscopies in children are carried out under GA. On account of the effects of anesthesia, pain during and after the procedure may not be appreciated well enough by the treating physician in the case of pediatric patients (25). However, studies carried out in adult patients have demonstrated that pain is significantly reduced with the use of CO_2_ insufflation during colonoscopy in sedated and non-sedated patients. Bretthauer et al. (30) in a study of 103 colonoscopy patients sedated with midazolam and pethidine reported a significant decrease in pain scores with CO_2_ insufflation at 1 and 3 h after the procedure. Similarly, Seo et al. (31) in a double-blind RCT on sedated patients undergoing colonoscopy have also reported a higher number of pain-free patients with CO_2_ as compared to air insufflation (91.6 vs. 76.1%). Another RCT has shown reduced pain scores with CO_2_ in non-sedated patients undergoing colonoscopy (32). The encouraging results in adults have prompted the use of CO_2_ in pediatric endoscopies as well, but, as seen in our review only five RCTs have been published to date. The results of our study concur with the outcomes reported in adult patients. On pooled analysis of data from 450 patients, our results indicated that CO_2_ insufflation can reduce the odds of postoperative pain by around 60% (95% CI: 13–81%). However, the strength of the evidence is reduced with the wide confidence intervals and the instability of the results on a sensitivity analysis. On the sequential exclusion of four of the five trials, the results were non-significant indicating no benefit of CO_2_ over air. This can be partly attributed to the heterogeneity in the included studies for the different age groups, different sedation protocols as well as the different scales used to evaluate pain in pediatric patients. It is known that pain scores in children can be very subjective and can depend upon the child's confidence to adequately deal with the pain (33). Furthermore, research also indicates that pain may not be a significant issue after GI endoscopy. Allen et al. (34) in a study of 227 patients have demonstrated that <50% of patients undergoing colonoscopy complain of pain and only 1/10th of all patients need an analgesic. Therefore, in the absence of pain, the utility of CO_2_ insufflation as compared to air may be questioned.

Pain-related to endoscopic procedures have been attributed to the overdistention of the bowel. Since CO_2_ undergoes rapid absorption via the intestinal mucosa and is quickly excreted via the respiratory tract, the use of this gas during endoscopic procedures can reduce post-operative abdominal distention (5, 6). Therefore, post-procedural abdominal girth can be an important surrogate marker in assessing the efficacy of CO_2_ insufflation in endoscopic procedures. Lack of adequate data prevented us from quantitatively examining post-procedural abdominal distention in our analysis. However, qualitative analysis revealed that only one trial reported significantly greater abdominal distention with air insufflation. It is important to note that abdominal girth was measured using tape in all studies. While adult studies have demonstrated lower abdominal distention using tape measurements, the technique is not considered to be accurate (31). Precise outcomes can only be assessed by radiological techniques but are limited due to ethical issues (23). Patient-reported bloating, though a subjective outcome, was reported to be lower with CO_2_ insufflation in two trials.

An important safety concern with the use of CO_2_ is the risk of systemic hypercarbia leading to cardiac or respiratory compromise (35). The rapid absorption of CO_2_ can potentially strain the respiratory system during its excretion. This may be further compounded by the use of sedation in pediatric patients leading to inadequate respiratory compensation (25, 36). Several RCTs in adults have therefore excluded patients with cardiac and respiratory illnesses, opioid users, and patients with high baseline pCO2 levels while assessing the efficacy of CO_2_ insufflation in GI endoscopies (32, 37). However, recent evidence shows that CO_2_ insufflation can be safely used in high-risk patients like those with obstructive ventilatory disturbance (38). Studies on healthy sedated adults have also shown CO_2_ insufflation to be safe (30, 31). In line with these studies, no safety concerns were reported with the use of CO_2_ in any of the included trials of our review. The significantly higher number of transient hypercarbia episodes in the study of Dike et al. (27) were all seen with upper GI endoscopies. The authors reported that these episodes were a result of belching of CO_2_ during EGD which were detected in exhaled breath. None of the trials reported abandoning the procedure due to high EtCO_2_ levels in any patient.

Our review has some limitations. Firstly, only five studies were available for inclusion. The sample size of three trials was <50 patients per group. Secondly, there were concerns of bias due to inadequate blinding, attrition, and selective reporting in some of the trials. This could have skewed outcomes in the review. Thirdly, there was inter-study heterogeneity amongst the included studies as mentioned earlier. Fourthly, lack of data, and standard reporting precluded us from assessing all outcomes via a meta-analysis. Pain was assessed only as a dichotomous variable and mean differences of pain scores at various time intervals could not be assessed.

Nevertheless, our study is the first review evaluating the efficacy of CO_2_ insufflation vs. air in pediatric GI endoscopies. A comprehensive literature search was conducted to extract all eligible studies. A descriptive analysis of all outcomes reported by the trials was performed to present complete evidence to the readers. A sensitivity analysis was also conducted to assess the influence of each study on the pooled effect size.

To conclude, our study indicates that the incidence of pain may be reduced with the use of CO_2_ insufflation in pediatric GI endoscopies without a significant risk of adverse events. However, current evidence is from a limited number of trials and not strong to recommend a routine of CO_2_ in pediatric gastroenterology practice. Further high-quality RCTs with a large sample size and evaluating standard outcomes on a common scale are required to supplement current evidence.

## Data Availability Statement

Publicly available datasets were analyzed in this study. This data can be found at: PubMed, Embase, Scopus, and CENTRAL.

## Author Contributions

CJ conceived and designed the study. XL and PH were involved in literature search and data collection. CJ and XL analyzed the data. CJ wrote the paper. PH reviewed and edited the manuscript. All authors read and approved the final manuscript.

## Conflict of Interest

The authors declare that the research was conducted in the absence of any commercial or financial relationships that could be construed as a potential conflict of interest.
